# Looking for more reliable biomarkers in breast cancer: Comparison between routine methods and RT-qPCR

**DOI:** 10.1371/journal.pone.0255580

**Published:** 2021-09-23

**Authors:** Emanuele Caselli, Cristina Pelliccia, Valeria Teti, Guido Bellezza, Martina Mandarano, Ivana Ferri, Kerstin Hartmann, Mark Laible, Ugur Sahin, Zsuzsanna Varga, Chiara Lupi, Fabrizio Stracci, Angelo Sidoni

**Affiliations:** 1 Department of Medicine and Surgery, Section of Anatomic Pathology and Histology, Medical School, University of Perugia, Perugia, Italy; 2 BioNTech Diagnostics GmbH, Mainz, Germany; 3 Institute for Pathology and Molecular Pathology, Universitätsspital Zürich, Zürich, Switzerland; 4 Umbria Cancer Registry, Perugia, Italy; 5 Department of Medicine and Surgery, Section of Public Health, University of Perugia, Perugia, Italy; Medical Faculty of Porto University and IPATIMUP, PORTUGAL

## Abstract

**Purpose:**

Decades of quality control efforts have raised the standards of immunohistochemistry (IHC), the principle method used for biomarker testing in breast cancer; however, computational pathology and reverse transcription quantitative PCR (RT-qPCR) may also hold promise for additional substantial improvements.

**Methods:**

Herein, we investigated discrepancies in the assessment of estrogen receptor (ER), progesterone receptor (PR), human epidermal growth factor receptor 2 (HER2) and marker of proliferation Ki67 comparing routinely obtained IHC (and FISH) data (ORI) with the results of manual (REV) and semi-automated (DIA) re-evaluation of the original IHC slides and then with RNA expression data from the same tissue block using the MammaTyper® (MT) gene expression assay.

**Results:**

Correlation for ER and PR was high between ORI IHC and the other three study methods (REV, DIA and RT-qPCR). For HER2, 10 out of 96 discrepant cases can be detected between ORI and REV that involved at least one call in the equivocal category (except for one case). For Ki67, 22 (29.1%) cases were categorized differently by either REV alone (n = 17), DIA alone (n = 15) or both (n = 10) and 28 cases (29.2%) for RT-qPCR. Most of the discrepant Ki67 cases changed from low to high between the original and following assessment and belonged to the intermediate Ki67 expression range (between 9 and 30%).

**Conclusions:**

Determination of the breast cancer biomarkers ER, PR, HER2 and Ki67 at the mRNA level shows high degree of correlation with IHC and compares well with correlations between original with subsequent independent manual or semi-automated IHC assessments. The use of methods with wider dynamic range and higher reproducibility such as RT-qPCR may offer more precise assessment of endocrine responsiveness, improve Ki67 standardization and help resolve HER2 cases that remain equivocal or ambiguous by IHC/FISH. In summary, our findings seem to configure RT-qPCR as a complementary method to be used in cases of either equivocal results or presenting, at the traditional determination assays, biomarkers expressions close to the cut-off values.

## Introduction

Analysis of breast cancer tissues by pathologists generates a wealth of information that can be used to classify disease for prognostic purposes and help guide therapeutic decisions. Recent molecular studies have lent further support to the historical importance of hormone receptors, growth factors and perturbations in proliferation as the key operators of breast cancer initiation and progression [1]. Thus, it is not surprising that assessing the expression of estrogen receptor (ER), progesterone receptor (PR), human epidermal growth factor two (HER2) and proliferation remains the cornerstone of breast cancer diagnosis and treatment [2], although the well-known problems of accuracy and reproducibility which have necessitated the issuing of various guidelines [3–6], are still persistent [7]. In fact, IHC is methodologically error-prone, as it is a multi-step process, highly exposed to variabilities of various kinds, such as from the use of non-standardized local practices or from different, non-equally performing albeit approved reagents [8, 9]. However, the main source of variation is linked to the intrinsic limits of immunohistochemical stains, both for marked influences of the pre-analytical phase [7] and the subjectivity of interpretative criteria [10] as well as extreme intra- and inter-tumoral heterogeneity [11]

Some IHC markers are more susceptible to methodological failures owing to these limitations. The proliferation marker Ki67 is the most prominent example, because it is expressed heterogeneously in tissues and is continuously distributed, which makes it difficult to identify a single, reproducible cut-off [10]. For HER2, IHC has been greatly improved by FISH. Unfortunately, FISH is a method that requires a specific expertise, a fluorescent microscope, and is more time-consuming. The complexities of FISH are exemplified in a recent guideline update, which aims to address an ever-expanding list of methodological intricacies [12]. However, there are other reliable methods of in situ hybridization for the assessment of HER2 amplification that surpass these drawbacks, such as CISH, SISH and DDISH.

Even though modern scientific advances were immensely assisted by corresponding technological breakthroughs, such as gene arrays and reverse transcription quantitative PCR (RT-qPCR), the landscape of breast cancer diagnostics remains dominated by conventional hematoxylin and eosin (H&E) stains and by immunohistochemistry (IHC) [13]. Current consensus does not favor the use of alternative methods such as for example RT-qPCR for assessing the expression of individual markers, and even though reliable and reproducible solutions are commercially available, their role however being mostly complementary [14, 15].

In 2017, the US Food and Drug Administration (FDA) announced the approval of the first whole slide imaging (WSI) system for primary diagnosis in surgical pathology [16]. Aside from RT-qPCR, automated digital image analysis (DIA) may also pave the way for more precise biomarker assessment and more accurate histopathological breast cancer diagnosis and has the potential to provide the objectivity and reliability required to transform biomarker testing in breast cancer. The entire process, however, involves several steps, including image acquisition, storage and management, annotation and viewing or sharing, each one requiring stringent quality control procedures [17].

Therefore, considering the diversity of candidate analytical methods and ongoing challenges with IHC/FISH, the decision for or against alternative methods for biomarker testing in breast cancer remains complex. In this respect, local attempts to benchmark new techniques against testing traditions may help estimate their feasibility and potential for routine use. In this study, we carried out parallel comparisons between 3 independent IHC assessments, including an image analysis solution and comparisons between these and RT-qPCR, using well-established local IHC methods, international scoring guidelines and the MammaTyper® gene expression assay. The aim of the study was to detect discordance between methods and discuss their clinicopathological implications.

## Material and methods

### Compliance with ethical standards

All procedures performed in studies involving human participants were in accordance with the ethical standards of the institutional and/or national research committee and with the 1964 Helsinki declaration and its later amendments or comparable ethical standards.

### Informed consent

The current study is a retrospective one, conducted on data and biological material already fully anonymized before the authors accessed them. The patients’ data and corresponding samples were accessed in the period between March 2010 and February 2012. According to the internal rules both of the University of Perugia and of the Hospital of Perugia, we have considered only the biological material in respect of which the patients expressed his/her specific informed consent to use such a material for research purposes; we also have observed all rules concerning confidentiality and protection of person data, in accordance with European Union, International and National rules.

### Patient selection

**Fig 1:**
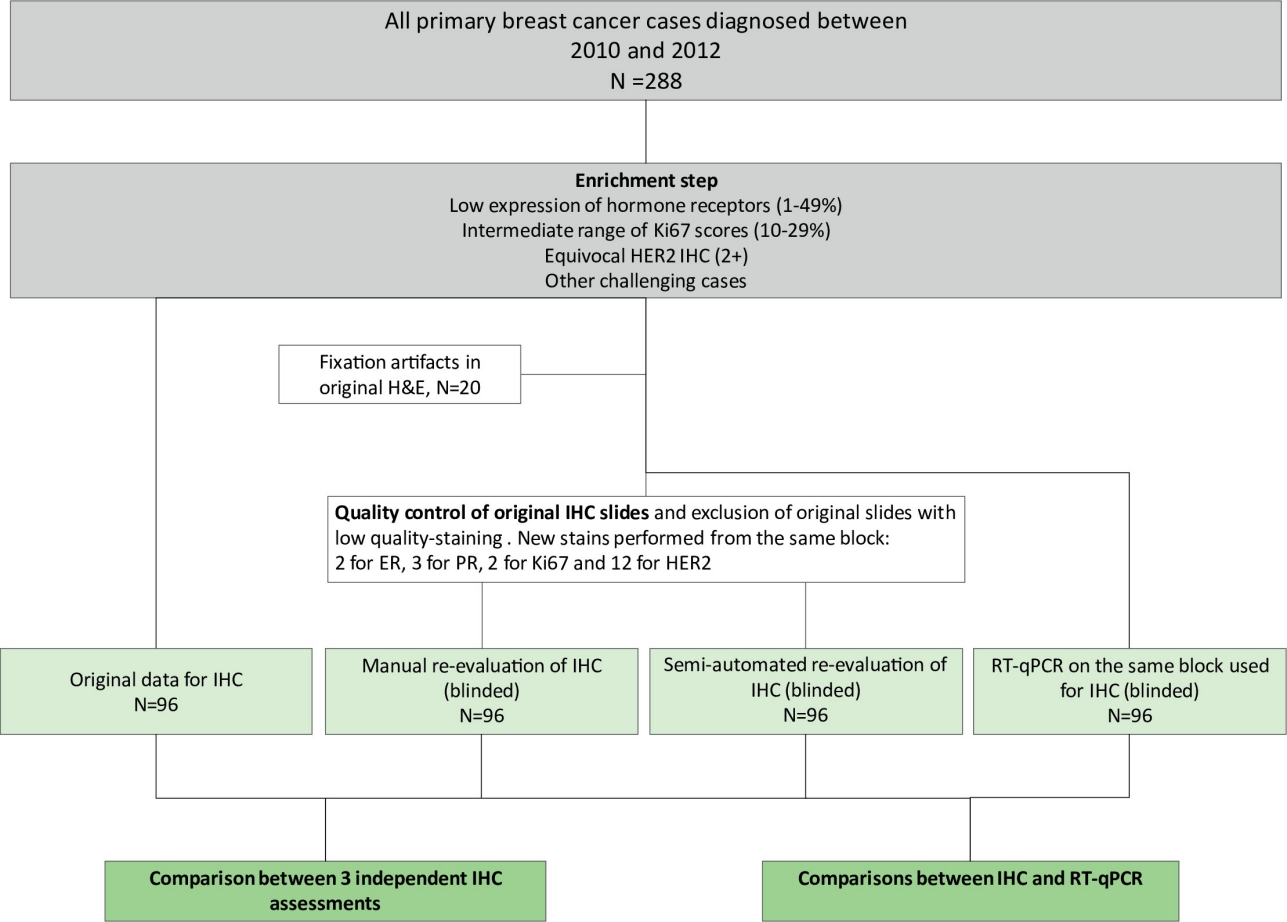
Study design.

Between March 2010 and February 2012, 288 women underwent surgery for invasive breast carcinoma (Section of Anatomic Pathology and Histology, Perugia, Italy). The IHC results from patient histopathology reports were screened to identify cases with varying levels of diagnostic difficulty (Fig 1). Available material from 116 cases, including FFPE blocks, H&E slides and IHC slides, were retrieved from the archive. The previously reported results for ER, PR, Ki67 and HER2 were registered as the original data (ORI) and consisted of assessments delivered during diagnostic routine by 6 trained pathologists according to available guidelines at that time [3, 6]. An experienced pathologist and FISH expert (GB) performed the ORI FISH data.

### Conventional histopathological assessments

The re-evaluation dataset (REV) was compiled as follows: An experienced pathologist (EC) that was not involved in the initial screening and was blinded to clinicopathological information carried out tumor classifications and grading on the available original H&E slides according to international standards in force at the time of the study [18, 19]. Twenty cases were excluded from further analyses due to inadequate fixation. In a second step the same pathologist re-evaluated ER, PR, Ki67 and HER2 on the existing IHC slides, but 2 cases for ER, 3 cases for PR, 2 cases for Ki67 and 12 cases for HER2 were re-stained, owing to missing original slides or low staining quality including fading. Standard international recommendations—in force when the research project was conducted, particularly according with the 2013 ASCO/CAP guidelines [4]—were used for the assessment and quantification of staining reactions [4, 6, 20]. Of note, original assessments for Ki67 were based on quick eyeballing, whereas formal counting was used upon re-evaluation. Due to the impossibility of re-evaluation of the original FISH slides, all HER2 equivocal cases by IHC (2+ score) after REV, regardless of ORI FISH data when available, were subjected to FISH analysis (dual-probe, Leica HER2 FISH system). Furthermore, we performed FISH analysis in all discrepant HER2/ERBB2 cases between IHC/FISH and MammaTyper®. Interpretation was performed by EC and an additional blinded evaluation was performed by GB in selected cases according to the ASCO/CAP 2013 guidelines (in force at the time of the study), either by counting signals in randomly chosen regions of interest with the assistance of a digital tool (Cytovision software, Leica Biosystems) (EC) or manually (GB). Of note, there was no disagreement between the two pathologists. Three selected cases with discrepancies to MammaTyper® ERBB2 determination were sent to an independent pathologist (ZV) for blinded FISH re-analysis (hybridization and evaluation).

### Computer-assisted IHC assessment

The same pathologist (EC) independently analyzed the expression of hormone receptors and Ki67 with a digital semi-automated image analysis (DIA) system (Aperio ImageScope, Leica Biosystems). All slides were scanned on the Aperio AT2 Scanner (Leica Biosystems) with a 40x magnification lens, using single focus layer without Z-stacking and were displayed on a 4K (ultra-high definition) monitor. On the digital slide (40x magnification), the pathologist selected randomly several regions of interest for ER and PR, whereas for Ki67 areas were chosen in such a way as to be representative of heterogeneity when present. Digital image analysis for assessing the percentage of positive nuclei was performed according to the Leica nuclear v9 algorithm. As regards HER2, the digital image analysis was not performed because of an optimized and reliable algorithm for assessing the membrane immunolabeling was unavailable at the time of the study.

### Immunohistochemistry (IHC)

All reactions were carried out on a BOND-III fully automated immunohistochemistry stainer (Leica Biosystems) with antibodies against ER, PR, HER2 and Ki67 according to vendor protocols and the use of appropriate positive and negative controls (Table 1).

**Table 1:** Immunhistochemistry staining specifications.

Antibody	Clone	Dilution	Manufacturer	Platform
**ER**	6F11	Ready to use	Leica Biosystems	BOND-III fully automated IHC
**PR**	16	Ready to use	Leica Biosystems	stainer (Leica Biosystems)
**Ki67**	MIB1	1:100	Dako	
**HER2**	Rabbit Anti-Human HER2 protein	Ready to use	Dako	BOND-III fully automated IHC
				stainer (Leica Biosystems) and AutostainerLink 48 Dako (for the 12 restained slides)

Of note, all 19 stains that had to be repeated were performed on the same block as the originals and without any reagent or protocol deviations compared to those available from the archive. Only for HER2, all slides prepared for re-evaluation were stained on the AutostainerLink 48(Dako) using Hercept Test™. Dewaxing and rehydration were performed manually. Antigen retrieval (pH = 6) was performed by heating the slides for 40 minutes at 97°C using the DakoPT-link.

### Fluorescence in situ hybridization (FISH)

FISH was carried out using the automated Leica HER2 FISH System on a BOND-III platform. Importantly, in the original determinations, FISH was performed using the HER-2 DNA ProbeKit II (PathVysion) with the Vysis Paraffin Pretreatment Reagent Kit. The dual-color probes identified the centromere 17 (Cep 17) as green signal, whereas the HER2/Neu gene/locus as orange one. Positive and negative controls were always available.

### RNA isolation and mRNA quantification with RT-qPCR

The MammaTyper® test was performed on 10 μm sections. In cases with tumor cell content <20%, manual dissection was conducted. MammaTyper® test was successfully performed in all 96 cases, applying manual dissection in 16 cases. Extraction of total RNA from FFPE samples was performed using a CE-marked paramagnetic bead-based method (RNXtract®, BioNTech Diagnostics, Mainz, Germany) according to the manufacturers’ instructions, as previously described in detail [21]. The expression levels of *ERBB2*, *ESR1*, *PGR* and *MKI67* were determined by RT-qPCR using the CE-marked MammaTyper® IVD kit (BioNTech Diagnostics) on the CFX96TM (BIO-RAD®) platform according to the manufacturer’s instructions. Calculations of delta-delta-Cq values were carried out as described previously [21], however data was further transformed to the relative unit scale (RU) to allow easier interpretation. The RU values is calculated by centering the delta-delta-Cq around the marker specific cutoff value. In this way, positive values correspond to gene expression higher than the cutoff and negative values represent an expression below the cutoff while the Cq (log2) scale is retained. Relative units (RU) = ((Median Cq target sample–Combined Reference sample)–(Median Cq target Positive Control–Combined Reference Positive Control))–marker specific cutoff. As cutoffs in this study, for ESR1 and PGR, a lower, diagnostic cutoff was applied (RU 0) which was established to fit the 1% IHC staining for ER and PR. For MKI67, the previously clinically validated cutoff was applied [22]. Rules for nominal categories (positive/negative) for all markers are summarized in Table 2.

**Table 2:** Instrument-specific relative units (RU) for CFX96^TM^ (BIO-RAD^®^) and the corresponding IHC equivalents.

RU	*ERBB2*			*ESR1*			*PGR*			*MKI67*	
	Negative	Equivocal	Positive	Negative	Low Positive	High Positive	Negative	Low Positive	High Positive	Low	High
**MammaTyper**	< -0.7	≥ -0.7 < 0	≥ 0	< 0	≥ 0 < 1.1	≥ 1.1	< 0	≥ 0 < 0.5	≥ 0.5	< 0	≥ 0
**IHC equivalent**	0 / 1+	2+	3+	0	1–49%	≥50%	0	1–19%	≥20%	<20%	≥20%

### Statistical analysis

The median difference, absolute median difference and standard deviation of the absolute median difference between ORI versus REV and ORI versus DIA for ER, PR and Ki67 were assessed. Calculation of intraclass correlation coefficient (ICC) which estimates the imprecision in relationship with the intersample variance was calculated using the formula: ICC = intersample variance/total variance [23, 24]. The Pearson correlation coefficient was used to measure the strength of the association between ORI versus REV, ORI versus DIA and ORI versus RT-qPCR for ER, PR and Ki67. Graphs were prepared using GraphPad Prism 6 (GraphPad Software, La Jolla, USA). A two-sided P value <0.05 was considered significant.

## Results

### Concordance between IHC assessments for ER, PR and Ki67

**Fig 2:**
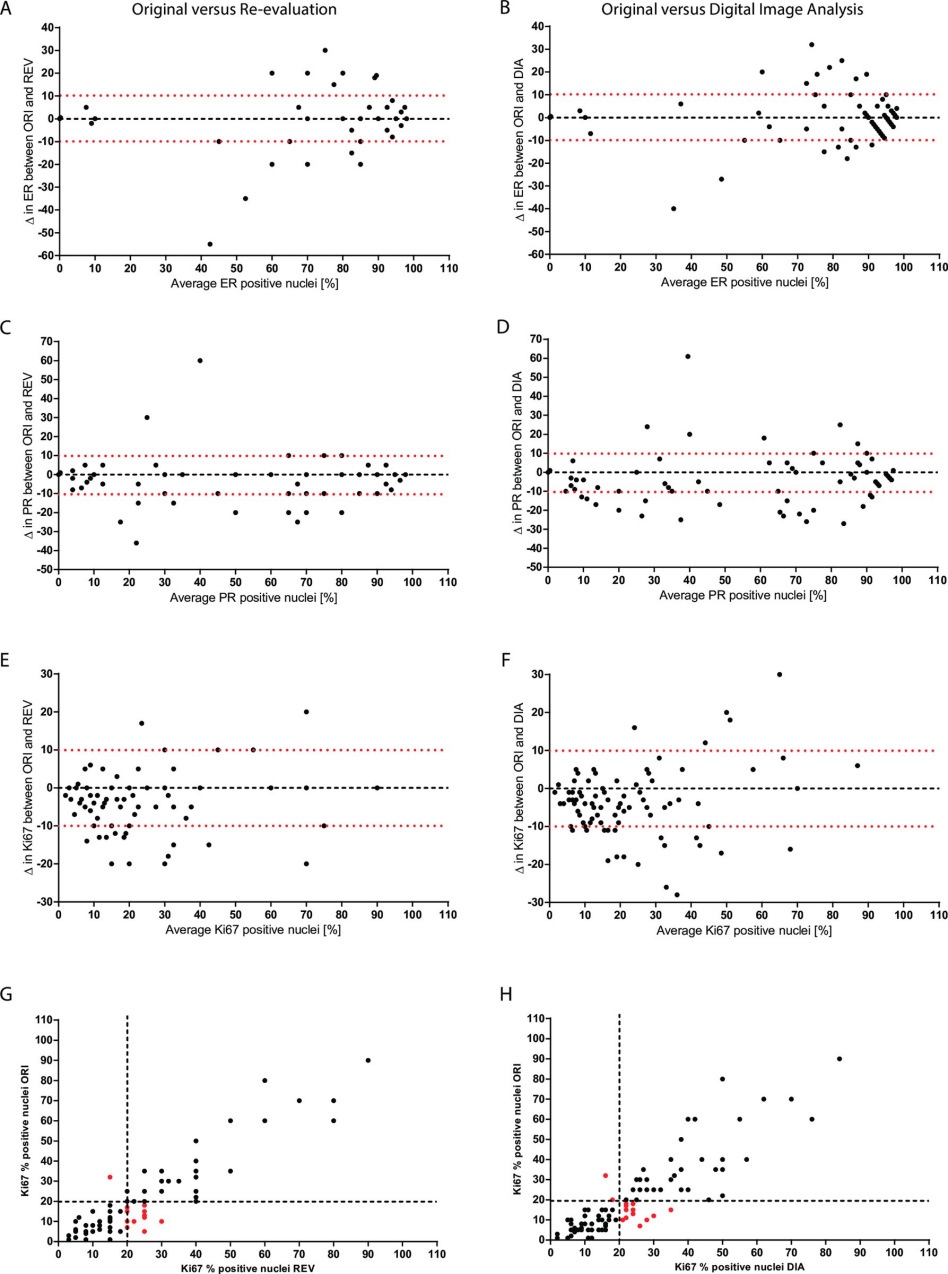
Correlation between original (ORI) and paired re-evaluated (REV) or digital image analyzed (DIA) samples for IHC. Bland-Altman plots showing differences between original, re-evaluated and digital image analysis for ER (A, B), PR (C, D) and Ki67 (E, F). Cut-off value at ±10% is shown by red dotted lines. Scatterplots showing correlation between original, re-evaluated and digital image analysis for Ki67 (G, H). Cut-off value at 20% for Ki67 is shown by black dotted lines. Red dots highlight discordant cases between original and either re-evaluated or digital image analysis.

The values for ICC shown in Table 3 indicate the high agreement between the continuous percentage scores of ORI IHC and the other two study methods (REV and DIA). There was no discordance for ER status between all three IHC assessments, with 10 ER-negative and 86 ER positive cases. Compared with original values, proportion scores differed in 58 cases upon REV (59.4%) and in 80 cases upon DIA (82.3%). In both cases, increase was more common than reduction (35 vs 23 for REV and 45 vs 35 for DIA), but in only 46 cases the change was in the same direction. Among 3 cases with a difference ≥ 25%, two belonged to the original group with positivity between 1–49%. Compared to ORI data, ER percentage positivity differed by more than 10% in 17 out of 96 cases (17.7%) for REV and in 22 out of 96 cases (22.9%) for DIA. Variabilities were further graphically analyzed with Bland-Altman plots (Fig 2A and 2B) and confirmed the trend of larger variations observed for samples with intermediate staining results (30–80% positive cells).

**Table 3:** Median proportion scores for IHC.

	ORI versus REV				ORI versus DIA			
	Median of Δ	Median of abs. Δ	SD of abs. Δ	ICC	Median of Δ	Median of abs. Δ	SD of abs. Δ	ICC
**ER**	0.0	3.0	8.6	0.949	0.0	4.0	7.4	0.955
**PR**	0.0	3.5	9.2	0.957	-2.5	5.0	9.3	0.945
**Ki67**	-3.5	5.0	5.6	0.903	-4.0	5.0	6.3	0.855

For ER, PR and Ki67 median and absolute (Abs.) median of difference between original and re-evaluation, as well as between original and digital image analysis was calculated. ICC: intraclass correlation.

Regarding PR, there were originally 18 negative and 78 positive cases with 2 discrepancies, both involving a difference between the original data and consecutive assessments, whereas the latter two were always concordant. Compared to original values, proportion scores differed in 57 cases upon REV (59.4%) and in 76 cases upon DIA (79.2%). In both cases, increase was more common than reduction (41 vs 16 for REV and 51 vs 25 for DIA), but in only 42 cases was the change in the same direction. Among 6 cases with a difference ≥ 25%, four belonged to the original group with positivity between 1–49%.

Compared to ORI data, PR percentage positivity differed by more than 10% in 30 out of 96 cases (31.3%) for REV and in 34 out of 96 cases (35.4%) for DIA (Fig 2C and 2D). As seen for ER, the largest differences in the staining percent assessments are seen in the lower range (15–80%).

There were 58 low and 38 high Ki67 cases, based on the original evaluation, but 22 (29.1%) were categorized differently by either REV alone (n = 17), DIA alone (n = 15) or both (n = 10). Most (twenty out of 22) discrepant cases changed from low to high between the original and following assessments (Fig 2G and 2H). Setting lower and upper Ki-67 cut-offs at 9% and 30%respectively to distinguish unequivocally low from high cases, we found that discrepant cases lied mostly between the two cut-offs, namely in the intermediate Ki67 expression range (median score of discrepant cases = 15%). In stark contrast, cases that were concordant between all 3 assessments, were either clearly low (median Ki67-low score = 5.5%) or clearly high (median Ki67-high score = 33.5%). As expected, discordance rates were significantly higher in the intermediate group (44.2%) compared with the low- and high groups (6.8% and 4.2% respectively). Compared to original values, proportion scores differed in 75 cases upon REV (78.1%) and in 94 cases upon DIA (97.9%). Upgrading was in both cases more common than downgrading (62 vs 21 for REV and 71 vs 23 for DIA) and was in the same direction in 63 cases. Discordant and concordant cases did not differ by any of the available clinic-pathological parameters. Compared to ORI data, Ki67 proportion scores differed by more than 10% in 26 out of 96 cases (27.1%) for REV and in 28 out of 96 cases (29.2%) for DIA (Fig 2E and 2F). With regard to absolute differences between the assessments, higher Ki67stainings (>15%) had clearly higher deviations than samples with a low Ki67 staining percentage (<15%).

### Correlation between IHC and RT-qPCR for ER, PR and Ki67

**Fig 3:**
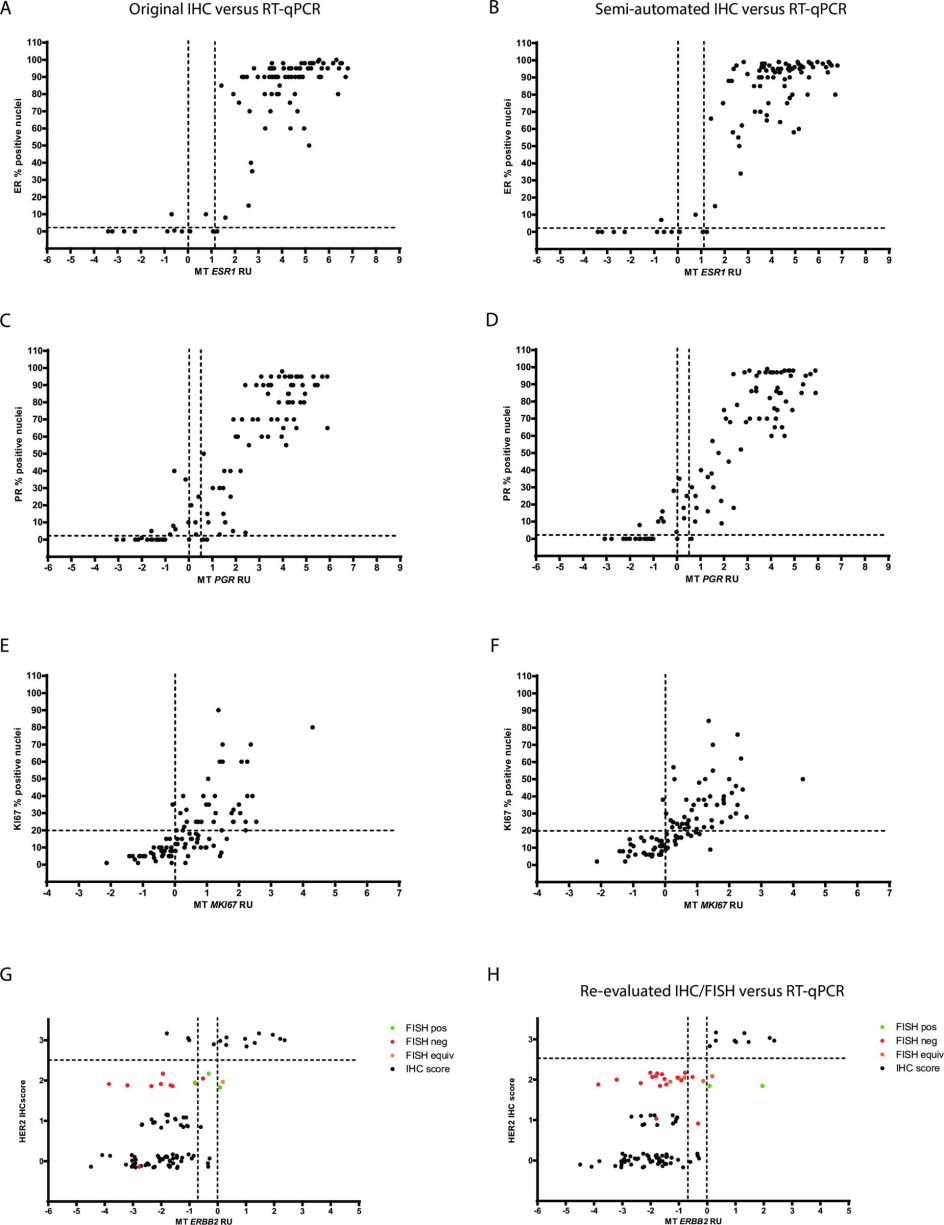
Comparison between IHC and RT-qPCR (MammaTyper®) for estrogen receptor (A, B), progesterone receptor (C, D), proliferation marker Ki67 (E, F) and human epidermal growth factor receptor 2 (G, H). Scatterplots with ORI IHC ER scores (A) or DIA IHC ER score (B) on the y-axis and *ESR1* RU values on the x-axis. Cut-off values at 1% for IHC and 0.0 RU for RT-qPCR are shown by black dotted lines. Scatterplots with ORI IHC PR scores (C) or DIA IHC PR score (D) on the y-axis and *PGR* RU values on the x-axis. Cut-off values at 1% for IHC and 0.0 RU for RT-qPCR are shown by black dotted lines. Scatterplots with ORI IHC Ki67 scores (E) or DIA IHC Ki67 score (F) on the y-axis and *MKI67* RU values on the x-axis. Cut-off values at 20% for IHC and 0.0 RU for RT-qPCR are shown by black dotted lines. Scatterplots showing correlation between IHC determination (ORI and REV) and RT-qPCR assessment for *ERBB2*/HER2 (G, H). Black dotted lines represent the respective cut-off for the RT-qPCR assay (*ERBB2*: 0.0) and for the positivity of the IHC staining (HER2: 2+). Second dotted line on the x-axis represents a second cut-off for ERBB2 (-0.7) after introduction of an equivocal zone.

Correlation between original IHC and RT-qPCR was very good for ER, PR and HER2 and good for Ki67 and strikingly comparable to the quality of the correlation between different IHC methods (Table 4). Estrogen receptor status by IHC (all 3 independent assessments) and MammaTyper® was discordant in 4 cases, with 3 out of 4 being negative at the protein level and positive at the RNA level (Fig 3A and 3B). By contrast, discordant cases for progesterone receptor were more often negative by RT-qPCR while being positive by REV or DIA IHC (7/9). The RNA expression of discordant cases took values close to the assay cut-off, while corresponding protein expression was low (Fig 3C and 3D). Two additional cases were discordant between MammaTyper® and the original assessment only.

**Table 4:** Agreement within IHC (ORI versus REV, ORI versus DIA) and between IHC (ORI) and RT-Qpcr.

	ER / ESR1			PR /*PGR*			Ki67 / *MKI67*		
**IHC cutoff**	Positive if ≥1%			Positive if ≥1%			Positive if ≥20%		
**RT-qPCR cutoff**	Positive if RU ≥ 0			Positive if RU ≥ 0			Positive if RU ≥ 0		
	ORI vs REV	ORI vs DIA	ORI vs RT-qPCR	ORI vs REV	ORI vs DIA	ORI vs RT-qPCR	ORI vs REV	ORI vs DIA	ORI vs RT-qPCR
**Pearson´s coefficient**	0.811	0.835	0.826	0.892	0.911	0.912	0.692	0.708	0.709

The discordance rate between the two methods for the proliferation marker was dependent on the type of IHC assessment being highest for the ORI data (29.2%), followed by comparable rates for DIA and REV (17.7% and 15.6% respectively). Among 32 discordant cases affected by discrepancies between RT-qPCR and any IHC assessment, 23 also displayed discordances between the different IHC data and 29 showed high MKI67 expression (Fig 3E and 3F). Discrepant cases had an average median Ki67 score of 16.7% compared to 25% in the concordant group and their RNA expression showed a tendency to cluster around the MKI67cut-off. Discordance was not associated with histology, grade or other available tumor characteristics.

### HER2 discrepancies

**Fig 4:**
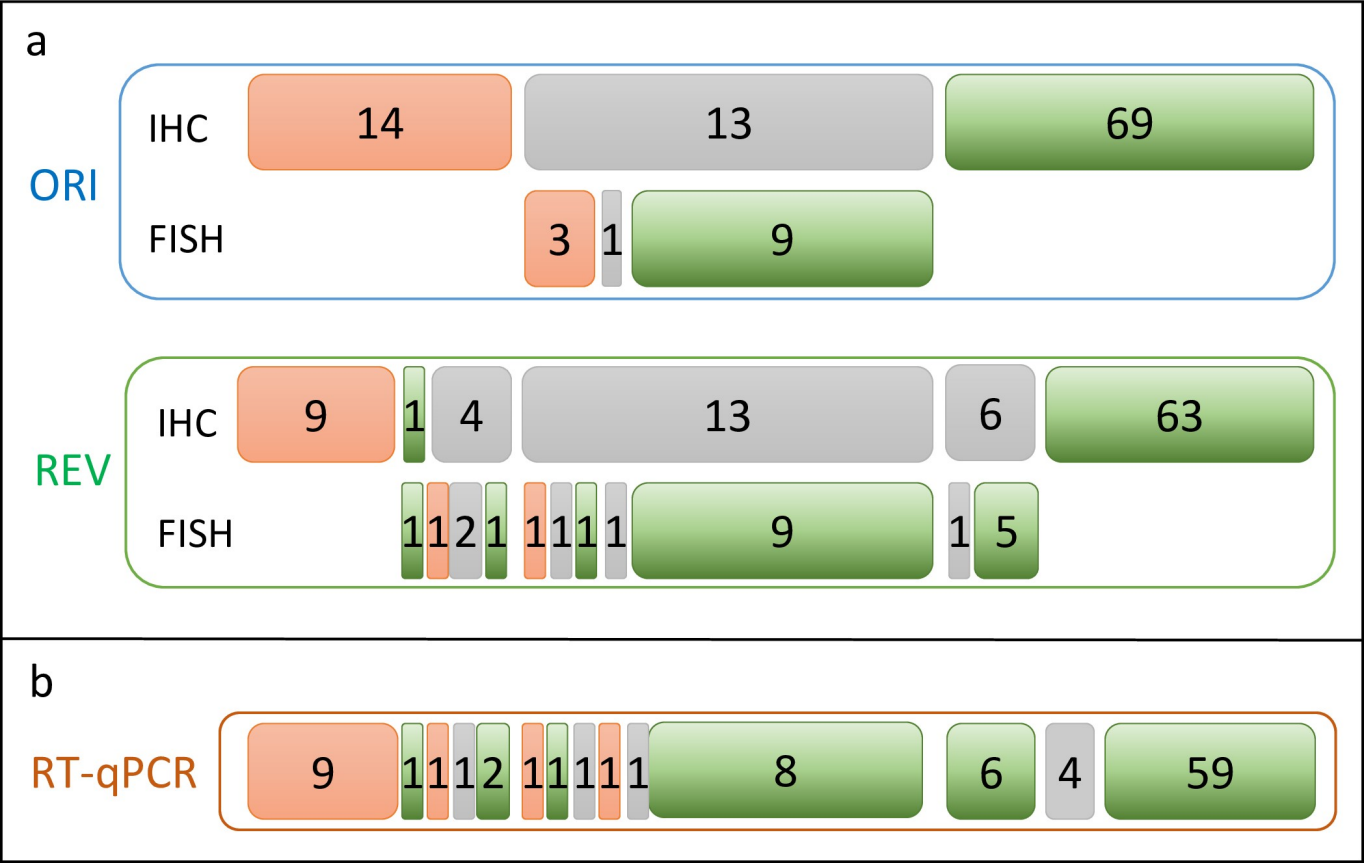
HER2 IHC/FISH discrepancies. Schematic analysis of discrepancies in HER2: a. between the original assessment (ORI) and re-evaluation (REV) and b. between IHC (ORI) and RT-qPCR. Positive calls are indicated by red filled boxes. Negatives calls are presented in green and equivocal calls in grey, according to the 2013 ASCO/CAP guidelines in force at the time of the study.

Based on the original combined IHC/FISH data, there were 17 positive cases for HER2. Except for one case, all other discrepancies involved at least one call in the equivocal category (2013 ASCO/CAP guidelines, which were employed at the time of the current study). Here, the REV FISH result was negative (in line with the RT-qPCR result and different than the ORI assessment that was positive). As shown in Fig 4A, re-evaluation identified 6 potential false positives. All 13 ORI HER2 equivocal cases remained equivocal upon REV, but REV identified 9 additional equivocal cases, 3 of which were ORI 3+, whereas 6 were ORI 0/1+. These 9 discrepant cases were subjected to FISH—evaluated according to the 2013 ASCO/CAP guidelines, which were in force at the time of the present study—, which agreed with the original IHC assessment in 6 cases. From the 14 cases with an ORI FISH result, FISH analysis was repeated for 13 samples. All 9 negative cases and 1 equivocal case were confirmed by re-evaluation. However, from the 3 ORI FISH positive results only one was confirmed in re-evaluation, while one was identified as negative and one as equivocal during the second round of re-evaluation by ZV. Furthermore, the blinded FISH re-analysis by ZV differed also in the 2 other cases tested: none case has been found amplified, but both were defined equivocal.

As shown in Fig 4B, discordance for HER2/ERBB2 was typically associated with cases being equivocal by at least one IHC assessment (ORI or REV). One ERBB2 potential false negative could be rescued by the introduction of an equivocal zone spanning 0.7 RUs to the left of the cut-off (Fig 3G and 3H).

### Impact of discordance on subtyping

**Fig 5:**
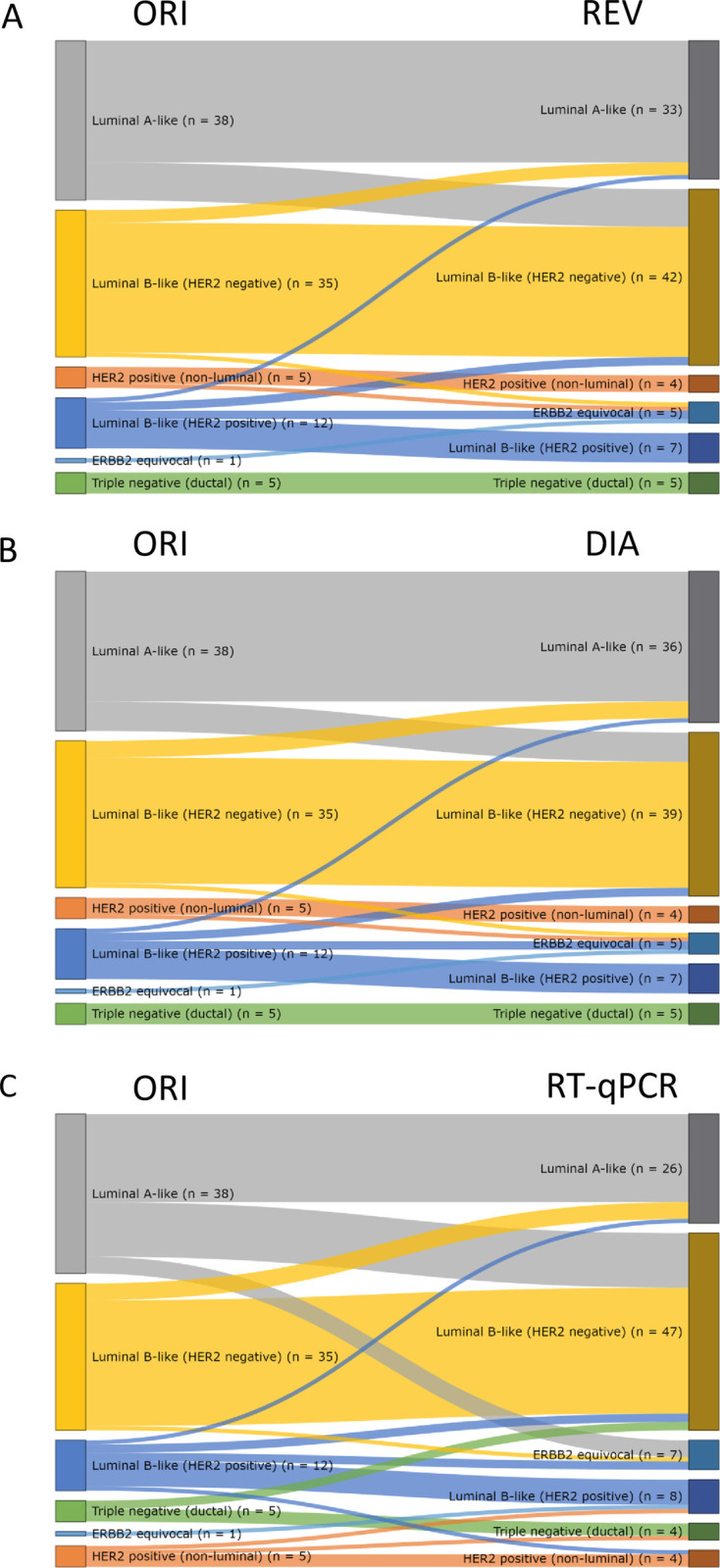
Subtype redistribution after re-evaluation (REV) (A), semi-automated analysis (DIA), (B) and RT-qPCR measurement (C).

Each tumor was assigned a molecular subtype according to the surrogate definitions of the St. Gallen expert panel [25, 26]. Not surprisingly considering the classification rules, the effect of discordance in the assessment of Ki67 was expressed as discrepancies mainly in the luminal categories with many originally Luminal A-like cases being subsequently categorized as Luminal B-like upon re-evaluation (23.7% for manual, 18.4% for digital) or when MammaTyper® RNA data were used instead of IHC (34.2%) (Fig 5).

Discrepant cases in the assessment of ER led to four luminal to non-luminal switches.

As regards HER2, the discrepancies highlighted by Mammatyper® allowed us to identify and better stratify 3 originally FISH-discrepant cases, overcoming the issues related to both laboratory quality assurance and inter-/intraobserver subjectivity in interpreting its immunohistochemical and ISH assessment.

## Discussion

Increasing the accuracy of breast cancer diagnosis to deliver the most precise and effective therapy is a continuous process involving on one end efforts to improve the performance of conventional methodologies and on the other end attempts to introduce technologically and operationally advanced analytical alternatives. The aim of our study was to explore discrepancies in the assessment of ER, PR, HER2 and Ki67 by comparing routinely obtained IHC (and FISH) data first with the results of manual and semi-automated re-evaluation of the original IHC slides and then with RNA expression data from the same tissue block using MammaTyper®. Immunohistochemistry showed a high degree of correlation for both ER and PR, even though the original data were generated by at least 6 different pathologists with variable experience in data interpretation, indicating the critical contribution of the 2010 scoring guidelines, used in both cases, in raising the standards of ER and PR testing by harmonizing interpretation. High correlation was also found between RT-qPCR and IHC; however, some cases were discordant as expected when comparing different methodologies and target molecules [27]. Notably, discrepant tumors expressed lower amounts of hormone receptor protein, while corresponding RNA levels were also near the cut-off probably the result of the association between weak (but not negative) biological signals and uncertainty of measurements. Discordance was overall somewhat higher for progesterone receptor, consistent with this marker’s higher quantitative variability [28]. Obviously, the bimodal frequency distribution of estrogen receptor contributed to the high degree of correlation between RNA and protein data, whereas the more heterogeneous natural distribution of progesterone receptor probably allows for more inter-section variability (which may have contributed to some of the discordance between IHC and RT-qPCR) eventually inflating the dependency of IHC assessments on rather perceptual and cognitive functions [29]. In fact, anecdotal evidence from daily pathology practice suggests that the concern over missed treatment opportunities prompts many pathologists to categorize breast tumors as ER-positive, even when staining of nuclei does not formally meet the 1% criterion, despite the fact that low ER and/or PR expression has repeatedly been linked to non-luminal biology and endocrine resistance [30–34]. Interestingly, most discrepancies for progesterone receptor in our study were scored low by IHC and were found negative at the RNA level, which is consistent with the view that, in tumors reacting weakly to antibodies against hormone receptors, gene expression profiles are indicative of more aggressive molecular subtypes [35]. These observations seem to confirm that the RNA testing utility better suited to quantitative biological continuity of hormone receptors, as advocated by some authors; however, the results about it were not always consistent [36–38].

Despite slight improvements, reliable assessment of Ki67 remains beyond reach in most parts of the world, owing mainly to the heterogeneity in the expression of this marker on tissue sections, which results in reduced reproducibility particularly in the mid-range of observations [39, 40]. Our data clearly suggest that guideline-driven formal counting of nuclei, either performed manually or with the help of imaging software, may result in considerable differences in Ki67 scores compared to quick “eyeballing”. Upon re-evaluation, the percentage of positive nuclei were assessed across several high-power fields accounting in this way for potential heterogeneity, especially from the so-called “hot-spots”, which may have been disregarded or preferentially targeted in the original assessment. Similarly, better averaging of RNA expression throughout the whole section may explain why MammaTyper® resulted in more MKI67-positive cases compared with IHC, a consistent finding in previous studies involving the assay [22]. Discrepancies may also be explained by the fact that the study pathologist was blinded to clinical data, whereas original assessments would be expected to have been far more biased, because, routinely, microscopic evidence is integrated with other available sources of information to reach an appropriate interpretation, including treatment preferences communicated over tumor boards [41, 42]. As expected, differences in Ki67 between original IHC data and MammaTyper® resulted in discrepancies in the St. Gallen classification, leading to a significant redistribution of the luminal subtypes in favour of the Luminal B-like category. Such redistributions were previously reported in the FinHer trial, in which MKI67 was superior to IHC in predicting patient outcome and detecting interactions between an RNA-based St. Gallen molecular classification and treatment [22]. Owing to the clinical diversity of the selected cases in this study, such effects were not investigated, as any associations between biomarker groups and outcomes would have been extremely difficult to interpret due to multiple confounding factors and limited events. As opposed to controversies surrounding the use of Ki67, IHC for HER2 supplemented by FISH to resolve equivocal cases consistently receive strong votes of confidence across most jurisdictions [13]. HER2 is a critical biomarker, signaling for an aggressive form of breast cancer and, when present in the tissues of patients, it allows them to access effective, but also potentially toxic treatment with HER2 blocking agents. Due to its importance, interpretation rules for HER2 IHC and FISH results are periodically revised to address persisting or newly identified challenges [3–5]. Meanwhile, experts remain reluctant to endorse new technologies, as intensive efforts for the standardization of existing methods have only recently started to payoff [8]. In our lab, the quality of HER2 testing improved upon the adoption of another analytical platform, which may explain discrepancies between original assessments and re-evaluation for cases that were re-stained with the current laboratory set-up that produces less background cytoplasmic staining. The use of the 2013 guidelines in the re-evaluation resulted in more equivocal cases, as has been already described [43]. Importantly, 3 FISH-positive cases with discrepant RT-qPCR results were tested independently and found to be equivocal, indicating that RNA may have helped identify potential false-positives by our own in-house FISH methodology. This finding is of importance, as it raises questions regarding the use of FISH as the “gold-standard” [44]. Although the present study presented a limitation having used outdated guidelines concerning the HER2 ISH interpretation [4] and the current recommendations [5, 12] do not longer provide an equivocal result for the ISH test, the RT-qPCR could be helpful in the interpretation of cases for which the second reader ISH analysis and the activation of the internal procedure to resolve the issue [5, 12] would nevertheless not reach a confident result.

Another solution for the standardization of breast cancer biomarkers and particularly for Ki67could be represented by automation with the help of image analysis platforms. In our study, performing DIA was labor-intensive, because regions of interest had to be manually selected due to insufficient performance of automatic detection of tumor nuclei; additional significant delays were caused by several software failures. In addition, we were not able to document any clear benefit from semi-automated scoring compared with manual re-evaluation. Nonetheless, recent studies have shown highly promising degree of reproducibility for computational scoring of Ki67 on TMAs or biopsies that outperforms conventional manual analyses even when the latter are carried out under highly standardized conditions [45–47]. Therefore, computational techniques may hold real potential for improving the reliability of quantitative determinations compared with the human eye despite shortcomings that affect routine usability. On the other hand, automated quantification of staining is similarly limited by the short dynamic range of chromogen-based IHC and thus the information that is harvested for prognostic or predictive purposes is less than what can be obtained by RT-qPCR [48]. Another significant aspect of the routine or investigational use of DIA is the sample drop-outrate. In our study, no test was lost neither by MammaTyper® nor by DIA; however, folding or twisting of tissue has been reported by others as a reason for considerable sample loss during automated analyses [49]. The decision between DIA and RT-qPCR for biomarker testing in breast cancer is further complicated by deployment conditions and pre-analytical requirements. At a first glance, RT-qPCR appears to represent a more realistic solution, due to savings in diagnostic time, broad accessibility of RT-qPCR instruments across laboratories, strong clinical validity and affordability. However, we are aware that the availability of these instruments is not widespread and, mainly for economic reasons, most of the pathology departments will continue to use immunohistochemistry to evaluate the expression of these biopathological parameters, obviously adhering carefully to external quality controls and specific guidelines. On the other hand, the coarseness of the cutoffs currently used could benefit from more refined methods of analysis, such as gene expression profiles.

Our study has some limitations. Although clinic-pathological data including follow-up data were available for all patients, we were unable to show meaningful associations between discordance and patient or tumor characteristics probably because of the highly heterogeneous nature of the cohort. For the same reason we reckoned that outcome statistics would be of limited value. In addition, sample size was a limiting factor necessitating random selection of approximately 100 cases among all those that initially fulfilled our selection criteria. Moreover, we did not calculate agreement between methods due to the highly artificial constellation of challenging and/or ambiguous cases, which would prevent any attempt to extend such findings to other clinical/diagnostic settings. Finally, the study used outdated guidelines for the interpretation of IHC and FISH results concerning HER2 evaluation, according to the recommendations in force at the time of the analysis.

In conclusion, the degree of correlation between IHC and RT-qPCR is high and compares well with the correlation between original with subsequent independent manual or semi-automated IHC assessments. Intrinsic marker properties such as the type of protein and RNA frequency distributions or spatial heterogeneity in whole sections may interact with interpretation bias to shape the extent of inter-observer or inter-method variability. The use of methods with wider dynamic range and higher reproducibility such as RT-qPCR may offer more precise assessment of endocrine responsiveness, improve Ki67 standardization and help resolve HER2 cases that present a difficult interpretation by IHC/FISH. In summary, our results seem to configure RT-qPCR as a complementary method to be used in all cases of equivocal results or close to the cut-off values.

## Supporting information

S1 Data (XLSX)

S2 Data (XLSX)
